# Effective knowledge translation approaches and practices in Indigenous health research: a systematic review protocol

**DOI:** 10.1186/s13643-017-0430-x

**Published:** 2017-02-20

**Authors:** Melody E. Morton Ninomiya, Donna Atkinson, Simon Brascoupé, Michelle Firestone, Nicole Robinson, Jeff Reading, Carolyn P. Ziegler, Raglan Maddox, Janet K. Smylie

**Affiliations:** 1 Well Living House, Li Ka Shing Knowledge Institute, St. Michael’s Hospital, 30 Bond Street, Toronto, Ontario M5B 1W8 Canada; 2 National Collaborating Centre for Aboriginal Health, 3333 University Way, Prince George, British Columbia V2N 4Z9 Canada; 3 CHAIM Centre, Carleton University, 1125 Colonel By Drive, Ottawa, K1S 5B6 Canada; 4 Dalla Lana School of Public Health, University of Toronto, Health Sciences Building, 155 College Street, 6th Floor, Toronto, M5T 3M7 Canada; 5 Canadian Partnership Against Cancer, 1 University Ave. Suite 300, Toronto, Ontario M5J 2P1 Canada; 6 First Nations Health Authority, 100 Park Royal S., West Vancouver, British Columbia V7T 1A2 Canada; 7 Simon Fraser University, Harbour Centre, 515 West Hastings Street, Vancouver, British Columbia V6B 5K3 Canada; 8 St. Paul’s Foundation, 1081 Burrard Street, Vancouver, British Columbia V6Z 1Y6 Canada; 9 Health Sciences Library, St. Michael’s Hospital, 30 Bond Street, Toronto, Ontario M5B 1W8 Canada; 10 University of Canberra, Canberra, Australia; 11 Department of Family and Community Medicine, St. Michael’s Hospital, 61 Queen Street East, Toronto, M5C 2T2 Ontario Canada; 12 Canadian Institutes of Health Research Applied Public Health Research Chair in Indigenous Health Knowledge and Information, Toronto, Canada

**Keywords:** Knowledge translation, Indigenous health, Knowledge sharing, Indigenous research, Indigenous knowledge, Systematic review

## Abstract

**Background:**

Effective knowledge translation (KT) is critical to implementing program and policy changes that require shared understandings of knowledge systems, assumptions, and practices. Within mainstream research institutions and funding agencies, systemic and insidious inequities, privileges, and power relationships inhibit Indigenous peoples’ control, input, and benefits over research. This systematic review will examine literature on KT initiatives in Indigenous health research to help identify wise and promising Indigenous KT practices and language in Canada and abroad.

**Methods:**

Indexed databases including Aboriginal Health Abstract Database, Bibliography of Native North Americans, CINAHL, Circumpolar Health Bibliographic Database, Dissertation Abstracts, First Nations Periodical Index, Medline, National Indigenous Studies Portal, ProQuest Conference Papers Index, PsycInfo, Social Services Abstracts, Social Work Abstracts, and Web of Science will be searched. A comprehensive list of non-indexed and grey literature sources will also be searched. For inclusion, documents must be published in English; linked to Indigenous health and wellbeing; focused on Indigenous people; document KT goals, activities, and rationale; and include an evaluation of their KT strategy. Identified quantitative, qualitative, and mixed methods’ studies that meet the inclusion criteria will then be appraised using a quality appraisal tool for research with Indigenous people. Studies that score 6 or higher on the quality appraisal tool will be included for analysis.

**Discussion:**

This unique systematic review involves robust Indigenous community engagement strategies throughout the life of the project, starting with the development of the review protocol. The review is being guided by senior Indigenous researchers who will purposefully include literature sources characterized by Indigenous authorship, community engagement, and representation; screen and appraise sources that meet Indigenous health research principles; and discuss the project with the Indigenous Elders to further explore the hazards, wisdom, and processes of sharing knowledge in research contexts. The overall aim of this review is to provide the evidence and basis for recommendations on wise practices for KT terminology and research that improves Indigenous health and wellbeing and/or access to services, programs, or policies that will lead to improved health and wellbeing.

**Systematic review registration:**

PROSPERO CRD42016049787.

**Electronic supplementary material:**

The online version of this article (doi:10.1186/s13643-017-0430-x) contains supplementary material, which is available to authorized users.

## Background

There are still large health disparities between Indigenous and non-Indigenous people in Canada and in many other countries worldwide [1, 2]. Research is well positioned to provide evidence of health disparities as well as test and evaluate approaches that can improve peoples’ health. In population health research, effective knowledge translation is critical to implementing program and policy changes that require shared understandings of knowledge systems, assumptions, and practices. The term *knowledge translation* (KT) is commonly used among health researchers and literature in Canadian contexts and is akin to terms used in other disciplines such as knowledge mobilization, synthesis, dissemination, transfer and exchange, knowledge to practice, and knowledge sharing [3–7]. Literature on KT emerged in the early 1990s in the Canadian context but used inconsistent terminology and conceptual frameworks for how knowledge is put into action [7, 8].

While terminology and concepts related to KT are constantly evolving, KT in Canadian health research contexts is often defined as “the process(es) through which knowledge is transformed into action” [9]. The term knowledge translation has been widely adopted, promoted, and prioritized by government funding agencies and research institutions as a way of making research visibly relevant and valuable [10]. In the context of Indigenous research in Canada, it has been suggested that appropriate KT is about sharing knowledge in ways that are “locally developed and contextualized” [11] and the pre-existing integration of knowing and doing that [Indigenous] people have been doing for a long time [12]. For this review, we are looking for KT that explicitly links knowledge (including Indigenous concepts of knowing and doing as being inseparable), learned from the research process or findings, to specific actions.

The impetus for this review was a recognition that mainstream KT initiatives and language do not necessarily resonate with Indigenous ways of knowing and doing. Indigenous scholars have critiqued mainstream KT as inadequate—inappropriate in some cases—for Indigenous health research contexts [9, 11, 13]. Mainstream concepts of KT are based on the premise that knowledge is acquired through ‘evidence-based’ research and that putting this knowledge into action *is* KT [14]. However, if KT is about sharing knowledge in contexts where the knowledge is both relevant and valued, KT may be inseparable from research itself in studies where researchers and research participants/informants exchange information throughout all research phases—from the development to the end-of-project phase. Indigenous views of what constitute knowledge, whose knowledge is shared with whom, how knowledge is shared, and in what contexts particular knowledge is relevant suggest that KT activities are inseparable from research activities.

Indigenous people have epistemologically and contextually specific health knowledge and practices that have been historically suppressed and ignored in research practices [15]. More specifically, mainstream research on Indigenous people has largely been void of culturally relevant, meaningful, engaging, contextual, or decolonizing knowledge. Instead of a colonial lens that assumes what research is ‘good’ for Indigenous people, decolonizing research with Indigenous people aims to develop, synthesize, and apply knowledge *within* Indigenous communities and contexts [15]. Smylie proposed that Indigenous KT in health research be defined as “Indigenously led sharing of culturally relevant and useful health information and practices to improve Indigenous health status, policy, services and programs” [12] or “sharing what we know about living a good life” [16].

While research practices, protocols, and approaches used in Indigenous contexts are improving, systemic and insidious inequities built into research institutions and funding agencies that inhibit Indigenous peoples’ control over, input into, and benefits from research remain [17, 18]. For example, mainstream research methodologies require specific types of adherence to and evidence of reliability and validity that are incompatible with (or exclude) Indigenous ways of knowing and doing [19]. The paucity of randomized controlled trials (RCTs) with Indigenous populations suggests that researchers are excluding Indigenous people from studies rather than considering the use of participatory research methods in RCTs that offer culturally relevant findings [20]. What counts as rigorous knowledge in Indigenous contexts is frequently at odds with mainstream concepts of knowledge, and furthermore, how knowledge is shared in Indigenous contexts is not necessarily valued, understood, or supported by research and funding institutions. Indigenous research, in contrast to mainstream research practices, requires researchers to self-locate^1^, openly share the purpose and motivation of a study, safeguard sacred Indigenous knowledge, have a decolonizing focus, and ensure community benefits through research [18, 19, 21, 22]. For research to adequately and respectfully engage and serve Indigenous peoples, we contend that wise and Indigenous research practices (including KT) must be better understood by non-Indigenous researchers, research institutions, research funders, and Indigenous communities engaged in research alike. The term *wise practices* is used to replace the terms *best* and *evidence*-*based practices* and reflect the inclusion of Indigenous knowledge and practices as core and robust sources of information. Madeleine Kētēskwew Dion Stout [23] argues that wise practices offer more than what many university-based and non-Indigenous researchers include as scientific evidence.

In Canada, researchers engaging with Indigenous peoples are expected to adhere to principles such as the four R’s of research—respect, reciprocity, relevance, and responsibility [24]—and the Ownership, Control, Access, and Possessions (OCAP®) principles [25], among other guidelines for Indigenous research. There is, however, little to no mention of such principles in mainstream KT activities or best practices to direct, or hold accountable, researchers in developing their KT plans. Many research institutions and non-Indigenous researchers conducting research involving Indigenous peoples remain unaware of how Indigenous principles differ from mainstream KT practices.

KT activities are widely endorsed and required in research designs. However, there is a dearth of published literature on how to practice, document, and evaluate Indigenous KT. In addition to conducting a preliminary search for documents that would meet our inclusion criteria, the research team reviewed two scoping reviews: (1) an unpublished paper on how KT is defined in Indigenous health research contexts [26] and (2) a paper on evaluated or assessed KT approaches in the Circumpolar North [27]. Published and grey literature draw attention to gaps between epistemic assumptions of Indigenous and non-Indigenous KT models and practices; however, there is no systematic review on the effectiveness of Indigenous KT in health research to date. Indigenous scholars have highlighted that KT strategies with Indigenous peoples must be re-conceptualized, further developed, and evaluated [9, 13, 15, 16, 28–30].

The aim of this review is to provide the evidence and basis for recommendations on wise practices for KT language and research in Canada (and abroad) to improve Indigenous health and wellbeing and/or access to services, programs, or policies that will lead to improved health and wellbeing. This systematic review aims to answer the question *what are the promising and wise practices for “knowledge translation” in the Indigenous health research field*? Identifying wise and promising practices will provide the basis for changes in research methods, knowledge, or circumstances that improve Indigenous wellbeing and determinants of health. This systematic review is also intended to eventually inform an Indigenous KT evaluation framework within Canada.

## Methods

This systematic review is guided by an advisory team that is comprised of five Indigenous and three non-Indigenous scholars with expertise in areas of Indigenous health and wellbeing, Indigenous knowledge, and KT in Indigenous contexts, in addition to an information specialist. All advisory team members were involved in the development of this protocol; at least two members will test the inclusion/exclusion criteria, quality appraisal tool, and data extraction tool; and all are listed co-authors for this paper.

### Literature search

We will search the indexed and non-indexed databases as well as online portals and repositories listed in Table 1. Major databases will be searched by a health information specialist research team member, using a comprehensive list of terms that identify Indigenous groups worldwide while the non-indexed databases and grey literature will be searched for Canadian studies. All documents from database inception to the present will be reviewed. The rationale for limiting the grey literature search to Canadian contexts is twofold: this systematic review is intended (1) to eventually inform an Indigenous KT evaluation framework within Canada and (2) to keep the systematic review manageable. The list of search terms to be used for indexed databases, non-indexed databases, online portals, and online repositories are listed in Table 2 (Additional file 1). To ensure that all relevant non-indexed grey literature reports are located, we will identify a minimum of three key informants at the federal level and each province/territory that fund research related to Indigenous health. Key informants will be contacted by email and, if required, a follow-up telephone call, to request any grey literature documents (such as reports or media files) that meet the inclusion criteria. We will track response rates and aim to have at least two informants from each province and territory respond.

**Table 1:** Search strategy

Databases	Aboriginal Health Abstract Database, Bibliography of Native North Americans, CINAHL, Circumpolar Health Bibliographic Database, Dissertation Abstracts, First Nations Periodical Index, Medline, National Indigenous Studies Portal, ProQuest Conference Papers Index, PsycInfo, Social Services Abstracts, Social Work Abstracts, Web of Science
Non-indexed and grey literature	Arctic Health Publications Database, Arctic Science and Technology Information System, CAMH Library, Canadian Best Practices Portal: Aboriginal Ways Tried and True, Canadian Health Research Collection, Canadian Knowledge Transfer and Exchange Community of Practice, Canadian Women’s Health Network, Centre for Indigenous Environmental Resources, First Nations Child & Family Caring Society of Canada, Google Scholar, Government of Quebec, Health Evidence, Hope-Lit Database, INSPQ: Public Health Expertise and Reference Centre, Inuit Studies, KT Clearinghouse, National Aboriginal Health Organization, National Collaborating Centre for Aboriginal Health, Native Health Database, Pan American Health Organization, Pimatisiwin, Population Health Improvement Research Network Library, University of Manitoba Health Sciences Libraries Aboriginal Health Collection
Indigenous health research funders	Aboriginal Affairs and Northern Development, Aboriginal Healing Foundation, Athabasca University: Indigenous Knowledge and Research, Canadian Institutes of Health Research: Institute for Aboriginal Peoples Health, Public Health Agency of Canada

**Table 2:** Search terms

Indexed database search terms	Comprehensive list of Indigenous groups globally, originally published by Kolahdooz et al. [35] and created from Food and Agriculture Organization of the United Nations [36] and Wikipedia’s List of Indigenous Peoples [37]. The Medline search strategies for Indigenous groups/identities, knowledge translation, and evaluation can be found in Additional file 1.
Non-indexed databases and grey literature	Indigenous, Aboriginal, Native, First Nation, Inuit, Métis, participatory action, knowledge translation, knowledge mobilization, knowledge, dissemination, evaluation, evidence

Search results will be imported into EndNote and uploaded to EPPI Reviewer, a software tool for the management of systematic reviews. Duplicates will be removed, and two reviewers will independently screen every title, abstract, and/or summary using four main inclusion criteria: (1) the research must be linked to Indigenous health and wellbeing; (2) the study must primarily be focused on Indigenous people; (3) the KT goals, activities, and rationale must be documented; and (4) the KT strategy must be evaluated and the outcome(s) identified (Table 3). All full texts of eligible screened citations will be reviewed by two independent reviewers to determine if they meet the inclusion criteria. Forward and backward citation searches of all included documents will be conducted to identify additional studies that also meet the review’s inclusion criteria. Disagreements between reviewers will be resolved through discussion or including a third reviewer (member of the research team) as required. First author [MEMN] will be a reviewer for both the title and abstract screening and the full text screening phases. The second reviewer will be an experienced and a trained Indigenous scholar or research assistant, well versed in concepts of Indigenous health and wellbeing and knowledge translation. Reviewers will check to ensure a minimum of 80% in agreement after screening 5% of the data before proceeding.

**Table 3:** Inclusion criteria

Studies	Research must be related to broad concepts related to Indigenous health and wellbeing.
Population	Research studies that include a majority of Indigenous people, excluding scoping/systematic reviews and guideline documents. Indigenous people can be from any geography and must be living with the effects of colonization.
Intervention	Documented knowledge translation goals, activities, and rationale using broad definitions/terminology for knowledge translation. We are, however, not including implementation science as a form of KT on the premise that it is focused on testing programs, tools, or practices and generating new data.
Outcome	Studied and/or evaluated formative or summative outcomes of knowledge translation work, including process-oriented evaluations

This systematic review will exclude studies that are not available in English and do not sufficiently meet the cutoff score of 6 or more (out of 12) using our quality appraisal tool.

### Quality appraisal

We will test and adapt the Well Living House quality appraisal tool [31, 32] intended for heterogeneous Indigenous studies that include qualitative and quantitative methodologies. This tool has specific questions under the three main categories of rigour of evaluation methods, strength of evidence, and relevance to the Indigenous community (Table 4). Two reviewers will independently appraise each document, answering the questions on a scale of 0–4 for each of the three domains, a maximum potential score of 12. The quality appraisal tool is intended to include methodologically heterogeneous studies that are strong in one of the three equally weighted domains and present evidence of addressing at least one other domain. In cases where there is insufficient or no information to assess a domain, we will contact the author to ask for further documentation to help complete the quality appraisal tool. The tool includes a community relevance domain to emphasize and include studies that explicitly and purposefully incorporate Indigenous knowledge systems and priorities. It is not required that studies score above zero in all three domains; reviewers will be assessing the appropriateness of the evaluation for an Indigenous context in two of the three domains.

**Table 4:** Well Living House quality appraisal tool

Category	Questions	Scoring criteria
Local community relevance of method and measures /4	1. Did the measures of success reflect local Indigenous community understandings of success?	(Yes = 2, Partial = 1, No = 0) Yes: evidence provided explicitly in the text (look for: where did evaluation take place, who collected evaluation data?) Partial: hints of including local community values/beliefs/knowledge systems in text and therefore assumption made by reviewers that evidence is present No: nothing was said or author(s) indicated that success was not defined by the community.
	2. Had methods and tools been tested and validated previously in a similar Indigenous context and reviewed for relevance by appropriate community members?	(Yes = 2, Partial = 1, No = 0) Yes: evidence is provided explicitly in text Partial: hints of using a tool that has been used in Indigenous contexts and therefore assumption made by reviewers that evidence is present No: nothing was said or author(s) said that the evaluation method/tool has not been used before in Indigenous contexts.
Rigour and internal validity of the evaluation method /4	3. Do the quantitative or qualitative methods meet relevant rigour and internal validity?	(Excellent = 4, Fair = 3, Barely Acceptable = 2, Poor = 1) Is the study design appropriate for evaluation research question(s)? Are the conclusions supported and justified by the results? Quantitative Is the sample size described and justified? Are the instruments/tools already validated? Are threats to validity addressed (such as confounding factors)? Qualitative Are the participants selected using appropriate strategies (such as purposive sample or until saturation is reached)? Is there accurate and adequate documentation of the KT effects described?
Strength of the evidence /4	4. Is the evidence strong?	(Excellent = 4, Fair = 3, Barely Acceptable = 2, Poor = 1) Quantitative Does the evidence have adequate power and statistical significance? Is the response rate reasonable? Qualitative Are there major and convincing themes from triangulation, and/or member checking?

Each of the domains will be scored by two independent reviewers, with expertise in Indigenous health and knowledge translation, one of whom is an Indigenous researcher. Documents that score 6 or more will be included in the data analysis. An inter-rater reliability test will be conducted at the beginning, mid-point, and end of the quality assessment stage.

### Data extraction

A data extraction tool will be developed and tested by three advisory team members using five randomly selected studies. Two reviewers, one of whom is an Indigenous scholar and both of whom have expertise in Indigenous health and KT, will independently extract data from each of the included articles. This process aims to ensure that systematic data extracted from each source will provide comprehensive data that can be used to synthesize the literature and inform best practices for KT in Indigenous health research contexts. The initial data extraction tool will be adapted from a scoping review of Inuit communities in the circumpolar regions [27].

### Data analysis

We will use a narrative synthesis [33] for methodologically strong and diverse studies that report on evaluated Indigenous health research KT. Narrative synthesis is an appropriate method for analysing a broad range of KT and evaluation methods and will be used to (a) produce rich descriptions of KT processes in Indigenous contexts, (b) identify themes and patterns across included studies, (c) describe the relevance of KT outcomes to Indigenous health and wellbeing and/or access to services, programs, or policies that will lead to improved health and wellbeing, and (d) identify KT evaluation methods and rationales.

## Discussion

Conventional systematic reviews on Indigenous health interventions have been critiqued as frequently being inappropriate and lacking practical relevance to or lacking engagement with Indigenous people and communities [34]. This systematic review, by design, departs from standard reviews in multiple ways. First, the review team is comprised of several senior Indigenous researchers with a career-long commitment to Indigenous community engagement and Indigenous health research. Second, the review purposefully includes a breadth of grey and non-indexed literature where there tends to be more Indigenous authorship, community engagement, and representation. Third, the screening and appraisal tools are being selected and adapted to meet Indigenous health research principles. Finally, the results from this review will be shared and discussed with the governing counsel of Indigenous grandparents at Well Living House (with St. Michael’s Hospital in Toronto), an action research centre dedicated to Indigenous health and wellbeing (www.welllivinghouse.com).

Recognizing that systematic reviews can be a means to inform policies and practices, the overall aim of this review is to provide the evidence and basis for recommendations on wise practices for KT language and approaches to health research that improve Indigenous health and wellbeing or services, programs, and policies that can improve Indigenous health and wellbeing. As a result, the systematic review findings will be of particular interest to knowledge users involved in research with Indigenous peoples, as well as stakeholders involved in Indigenous program and policy development, implementation, and evaluation. Complementary to this systematic review, we will also engage with Indigenous Elders to better understand what it means to draw on and share Indigenous knowledge. A key Indigenous research principle in Canada is that researchers must meaningfully engage with Indigenous people to ensure Indigenous knowledge is shared in a respectful way and is shared with context provided (of Indigenous knowledge, how it was shared, and with what intent). Including Indigenous Elders in research focused on Indigenous knowledge and KT in Indigenous contexts is essential to providing context for an Indigenous KT evaluation framework.

The knowledge sharing and dissemination plan for this systematic review includes, but is not limited to, academic publications, an accessible summary report with visual graphics, presentations and facilitated discussions in national and international forums, and conference presentations. As the systematic review process is underway, additional knowledge sharing mechanisms will be discussed and initiated.

### Strengths and limitations

This review also offers many strengths, including input and participation from Indigenous people at all stages of the research process which adds culturally relevant insight and context to a limited evidence base. This study will provide novel ideas of how to conceive of, practice, and align the overall aim of KT—to ‘make research matter’—with Indigenous ways of knowing in doing. In doing so, Indigenous peoples’ health and wellbeing has a greater chance of improving and benefiting from Indigenous health research. This research is subject to some limitations. Firstly, grey literature will be limited to Canadian contexts. Secondly, unfamiliar terms may be missed since language around KT varies through time and geography. Thirdly, community-based participatory research (CBPR) practices sometimes incorporate integrated KT approaches where knowledge is shared/co-produced and used throughout the research project. It is possible that this review will miss evaluated components of CBPR studies that address KT but use terms not included in our search strategy.

## Additional file

Additional file 1: Medline Search Strategy. As the title suggests, it is the complete search strategy used for Medline. (PDF 101 kb)

## Abbreviations

CBPR: Community-based participatory research
KT: Knowledge translation

## Footnotes

1 Self-location is essentially sharing who we are, where we come from, and our relation to an identity/place that informs how we lived in the past and the present.
