# Supplementary material for: Effective knowledge translation approaches and practices in Indigenous health research: a systematic review protocol
Source: Syst Rev. 2017 Feb 20;6:34. doi: 10.1186/s13643-017-0430-x (PMC5319191; doi:10.1186/s13643-017-0430-x)
Supplement: Additional file 1: — Medline Search Strategy. As the title suggests, it is the complete search strategy used for Medline. (PDF 101 kb) [file 13643_2017_430_MOESM1_ESM.pdf]

APPENDIX 1
Effective knowledge translation approaches and practices in Indigenous health research: A systematic review protocol

Medline Search Strategy

Database: Ovid MEDLINE: Epub Ahead of Print, In-Process & Other Non-Indexed Citations, Ovid MEDLINE® Daily and Ovid MEDLINE® <1946-Present>
Search Strategy:
----

1 american native continental ancestry group/ or indians, north american/ or inuits/
2 indians, central american/
3 indians, south american/
4 Oceanic Ancestry Group/
5 United States Indian Health Service/
6 Health Services, Indigenous/
7 (Aborigin* or Indigenous or Eskimo* or Inuit* or Inuk* or Metis or First Nations or First Nation or 1st nation or 1st nations or Native Canadian* or Native American* or Maori* or Pacific Islander* or American Indian* or Amerindian* or Native Alaska* or Alaska Native* or Native Hawaiian* or Torres Strait Islander* or on-reserve or off-reserve or tribal or autochtone* or amerindien* or indigene*).tw,kw.
8 (indian or indians).tw,kw.
9 India/ or India.tw,kw. or India's.tw,kw.
10 8 not 9
11 1 or 2 or 3 or 4 or 5 or 6 or 7 or 10
12 Pygmy peoples.tw. [Central Africa]
13 (Acholi or Alur or Ambo or Ankole or Antalote or Aushi or Aweer or Babongo or Baganda or Bahima or Ankole or Bagisu or Bagwere or Bakiga or Bakonjo or Basoga or Batoro or Bemba or Betsileo or Bisa or Bunyoro or Cafre or Chagga or Chewa or Chikunda or Chokwe or Chopi or Cishinga or Gova or Hadzabe or Haya or Hehe or Hutu or Ila or Inamwanga or Iteso or Iwa or Jopadhola or Kabende or Kalenjin or Kamba or Kaonde or Karamojong or Kikuyu or Kisii or Kosa or Kunda or Kwandi or Kwandu or Kwangwa or Lala or Lamba or Lango or Lenje or Leya or Lima or Liyuwa or Lomwe or Lozi or Luano or Lucazi or Lugbara or Luhya or Lumbu or Lunda or Lundwe or Lungu or Luo or Luvale or Luunda or Maasai or Makoa or Makoma or Makonde or Makua or Mambwe or Manyika or Mashasha or Mashi or Mbowe or Mbukushu or Mbumi or Mbunda or Mbwela or Merina or Meru or Mukulu or Mulonga or Munyoyaya or Ndau or Ndembu or Ng'umbo or Ngonde or Ngoni or Nguni or Nkoya or Nsenga or Nyakyusa or Nyamwezi or Nyanja or Nyengo or Nyiha or Ogiek or Oimatsaha or Pare or Rundi or Rwanda or Sakalava or Seba or Sena or Senga or Sengwer or Shangana or Shanjo or Shila or Shona or Simaa or Soli or Subiya or Sukuma or Swaka or Swahili or Tabwa or Tambo or Toka or Totela or Tumbuka or Tonga or Tutsi or Twa peoples or Unga or Wandya or Watha or Yao or Yiaku or Yaaku or Yombe or Zulu).tw. [East Africa]
14 (Afar or Agaw or Akisho or Anuak or Beja or Berta or Bilen or Borana Oromo or Daasanach or Dorze or Gumuz or Gurage or Hamar or Hedareb or Jeberti or Kichepo or Kunama or Me'en or Mursi or Nara or Nuer or Nyangatom or Oromo or Rashaida or Rer Bare or Saho or Shanqella or Sidama or Silt'e or Somalis or Suri or Tigre or Tigray-Tigrinya people or Tigrinya or Tigray-Tigrinya people or Tigray or Tirma or Welayta or Zay).tw. [Horn of Africa]
15 (Berber or Amazigh or Dinka or Egyptians or Haratin or Nuba or Nubians or Nuer or Shilluk or Tuareg).tw. [North Africa]
16 (Bushmen or Khoikhoi or Namaqua).tw. [Southern Africa]
17 (Baka or Balengue or Benga or Bubi people or Bujeba or Combe or Ndowe or Duala people or Beti-Pahuin or Ogoni people or Serer people or Tuareg or Toubou or Igbo).tw. [West Africa]
18 (Carib or Galibi or Taino or Neo-Taino or Ciboney or Ciguayo or Lucaya or Macorix or Guanahatabey or Eyeri).tw. [The Caribbean]

19 (Achi or Bokota or Bribri or Garifuna or Chorotega or Chorti or Chuj or Embera or Guaymi or Itza or Ixil or Jakaltek or Tolupan or Kaqchikel or Kiche or Kuna or Lenca or Maleku or Mam or Miskito or Mopan or Maya or Naso or Ngobe Bugle or Pech or Poqomchi or Poqomam or Qanjobal or Qeqchi or Rama Nicaragua or Sumo or Tojolabal or Tzutujil or Xinca).tw. [Central America]

20 (First Nations or Cree or Inuktitut or Ojibway or Innu or Dene or Oji-Cree or Anishinini or Mikmaq or Dakota or Sioux or Atikamekw or Blackfoot or Inuit or Metis or Nuxalk).tw. [Aboriginal Canadians]

21 (Alaska Athabascan or Ahtna or Deg Hitan or Denaina or Gwichin or Han or Holikachuk or Kolchan or Koyukon or Lower Tanana or Tanacross or Upper Tanana or Eyak or Haida or Tlingit or Tsimshian or Eskimo or Inupiat or Yupik or Cupik or Sugpiaq or Alutiiq or Alutiqu or Chugach or Koniag or Aleut or Unangan).tw. [Alaska Natives]

22 (Bannock or Coso People or Kawaiisu or Mono or Timbisha or Panamint or Koso or Washo or Palagewan or Pahkanapil or Kucadikadi or Owens Valley Paiute or Chemehuevi or Kaibab or Kaiparowtis or Moapa or Panaca or Panguitch or Paranigets or Shivwits or Guchundeka or Kuccuntikka or Tukkutikka or Tukudeka or Bohoinee or Pohoini or Pohogwe or Sage Grass people or Sagebrush Butte People or Agaideka or Doyahinee or Kammedeka or Kammitikka or Hukunduka or Tukudeka or Dukundeka or Yahandeka or Yakandika or Kusiutta or Goshute or Gosiute or Kuyatikka or Kuyudikka or Mahaguaduka or Painkwitikka or Pasiatikka or Tipatikka or Tsaiduka or Tsogwiyuyugi or Waitikka or Watatikka or Wiyimpihtikka or Capote or Moanunts or Muache or Pahvant or Sanpits or Timpanogots or Uintah or Uncompahgre or Taviwach or Weeminuche or White River Utes or Parusanuch or Yampa).tw. [Indigenous peoples of the Great Basin]

23 (Anishinaabe or Anishinape or Anicinape or eshnabe or Nishnaabe or Saulteaux or Nakawe or Apache or Lipan or Kiowa Apache or Arapaho or Arapahoe or Besawunena or Nawathinehena or Arikara or Arikaree or Arikari or Atsina or Gros Ventre or Blackfoot or Kainai Nation or Northern Peigan or Aapatohsipikani or Blackfeet or Siksika or Cheyenne or Suhtai or Comanche or Plains Cree or Absaroka or Apsaalooke or Escanjaques or Hidatsa or Ioway or Kansa or Kanza or Kiowa or Mandan or Missouria or Omaha or Osage or Otoe or Oto or Pawnee or Chaui or Kitkehakhi or Pitahawirata or Skidi or Ponca or Quapaw or Sioux or Santee or Yankton or Yanktonai or Lakota or Teton or Sichangu or Brule or Oglala or Itazipcho or Hunkpapha or Hunkpapa or Mnikhowozu or Miniconjou or Sihasapa or Oohenunpa or Nakoda or Nakota or Teyas or Tonkawa or Tsuu Tina or Wichita or Kichai or Rayados or Taovayas or Tawakoni or Waco).tw. [Plains Indians]

24 (Abenaki or Tarrantine or Kennebec or Caniba or Anishinaabe or Anishinape or Anicinape or Neshnabe or Nishnaabe or Algonquin or Nipissing or Ojibwa or Saulteaux or Nakawe or Odawa people or Potawatomi or Assateague or Attawandaron or Beothuk or Choptank people or Conoy or Erie or Etchemin or Meskwaki or Ho-Chunk or Winnebago or Honniasont or Illiniwek or Cahokia or Kaskaskia or Miami or Mitchigamea or Moingona or Peoria or Tamaroa or Wea or Haudenosaunee or Cayuga or Mohawk or Oneida or Onondaga or Seneca or Mingo or Tuscarora or Kickapoo or Laurentian or Lenni-Lenape or Munsee Esopus or Waoranecks or Warranawankongs or Minisink or Ramapough Mountain Indians or Unami or Acquackanonk or Hackensack or Navasink or Raritan or Rumachenanck or Haverstraw or Tappan or Unalachtigo or Wiechquaeskecks or Mascouten or Massachusett or Ponkapoag or Menominee or Mahican or Housatonic or Mahican or Wappani or Wappinger or Wappinger proper or Hammonasset or Kitchawank or Kichtawanks or Kichtawank or Mattabesset or Massaco or Menunkatuck or Nochpeem or Paugusset or Podunk or Poquonock or Quinnipiac or Eansketambawg or Rechgawawanc or Recgawawanc or Sicaog or Sintsink or Siwanoy or Tankiteke or Tunxis or Wecquaesgeek or Wyachtonok or Massachusett or Mikmaq or Micmac or Mohegan or Montaukett or Montauk or Nanticoke or Narragansett or Niantic or Nipmuc or Nipmuck or Occaneechee or Passamaquoddy or Patuxent or Penobscot or Pequot or Petun or Tionontate or

Pocumtuc or Poospatuck or Quapaw or Quinnipiac or Hammonasset or Mattabesec or Mattatuck or Menunkatuck or Meriden or Mioonkhtuck or Naugatuck or Nehantic or Paugusset or Podunk or Potatuck or Totoket or Tunxis or Wangunk or Wepawaug or Sauk or Shawnee or Shinnecock or Susquehannock or Tauxenent or Doeg or Unquachog or Wampanoag or Nauset or Patuxet or Pokanoket or Wawenoc or Wenro or Wenrohronon or Wicocomico or Wolastoqiyik or Wyandot).tw. [Indigenous peoples of the Northeastern Woodland]

25 (Chinook peoples or Cathlamet or Clackamas or Clatsop or Kathlamet or Multnomah or Wasco-Wishram or Watlata or Interior Salish or Chelan or Coeur dAlene Tribe or Entiat or Flathead or Selisch or Salish or Kalispel or Pend dOreilles or Methow or Nespelem or Nlakapamux or Thompson people or Nicola people or Okanagan or Secwepemc or Shuswap people or Sinixt or Sinkiuse-Columbia or Spokane people or Statimc or Lilwat or In-SHUCK-ch or Wenatchi or Wenatchee or Sanpoil or Sinkayuse or Sahaptin people or Upper Cowlitz or Taidnapam or Kittitas or Upper Yakima or Klickitat Tribe or Nez Perce or Pshwanwapam or Pswanwapam or Skinpah or Tenino or Warmsprings or Tygh or Upper Deschutes or Umatilla or Walla Walla or Wanapum or Wauyukma or Wyam or Lower Deschutes or Yakama or Cayuse or Celilo or Wayampam or Cowlitz or Fort Klamath or Kalapuya or northwest Atfalati or Tualatin or Mohawk River or Santiam or Yaquina or Kutenai or Kootenai or Ktunaxa or Lower Snake people or Chamnapam or Wauyukma or Naxiyampam or Modoc or Molala or Molale or Palus or Palouse or Upper Nisqually or Mishalpan).tw. [Indigenous peoples of the Northwest Plateau]

26 (Tlingit or Nisgaa or Tsetsaut or Haida or Tsimshian or Gitxsan or Haisla or Heiltsuk or Wuikinuxv or Kwakwakawakw or Nuu-chah-nulth or Makah or Coast Salish or Nuxalk or Willapa or Chimakum or Quileute or Chinook).tw. [Indigenous peoples of the Pacific Northwest Coast]

27 (Abihka or Acolapissa or Colapissa or Ais or Alabama or Alafay or Alafia tribe or Pojoy or Pohoy or Costas or Alafeyes or Alafaya Costas or Amacano or Apalachee or Apalachicola or Atakapa or Akokisa or Bidai or Deadose or Orcoquiza or Patiri or Tlacopsel or Avoyel or Backhooks Nation or Chuaque or Holpaos or Huaq or Nuaq or Pahoc or Pahor or Paor or Uca or Bayogoula or Biloxi or Boca Ratones or Caddo Confederacy or Adai or Adaizan or Adaizi or Adaise or Adahi or Adaes or Adees or Atayos or Cahinnio or Doustioni or Eyeish or Hais or Hainai or Hasinai or Kadohadacho or Nabedache or Nabiti or Nacogdoche or Nacono or Nadaco or Nanatsoho or Nasoni or Natchitoches or Neche or Nechaui or Ouachita or Tula or Yatasi or Calusa or Cape Fear Indians or Catawba or Esaw or Usheree or Ushery or Yssa or Chacato or Chakchiuma or Chatot or Chacato or Chactoo or Chawasha or Washa or Cheraw or Saura or Cherokee or Chickamauga or Chiaha or Chickahominy or Chickanee or Chiquini or Chickasaw or Chicora or Chine or Chisca or Cisca or Chitimacha or Choctaw or Houma or Chowanoc or Creek or Congaree or Canggaree or Coree or Coushatta or Coharie or Cusabo or Eno people or Garza or Grigra or Gris or Guacata or Santaluces or Guacozo or Guale or Cusabo or Iguaja or Ybaja or Guazoco or Hitchiti or Hooks Nation or Chuaque or Huaq or Nuaq or Jaega or Jaupin or Weapemoc or Jobe or Hobe or Jororo or Keyauwee or Koasati or Coushatta or Koroa or Luca people or Lumbee or Mabila or Mobile or Movila or Machapunga or Manahoac or Mattaponi or Matecumbe or Matacumbses or Matacumbe or Matacombe or Mayaca people or Mayaimi or Mayami or Mayajuaca or Meherrin or Mikasuki or Miccosukee or Mocoso or Monetons or Monyton).tw. [Indigenous peoples of the Southeastern Woodlands]

28 (Monekot or Moheton or Mougoulacha or Muscogee or Nahyssan or Naniaba or Nansemond or Natchez or Neusiok or Newasiwac or Neuse River Indians or Nottaway or Occaneechi or Siouan or Oconee people or Ofo or Okchai or Ogchay or Okelousa or Opelousas or Osochee or Oswichee or Usachi or Oosecha or Pacara or Pakana or Pacani or Pagna or Pasquenan or Pak-ka-na or Pacanas or Pamlico or Pamunkey or Pascagoula or Patiri or Pee Dee or Pedee or Pensacola or Potoskeet or Quinipissa or Rappahannock Tribe or Saluda or Saludee or Saruti or Santee or Seretee or Sarati or Sati or Sattees or Santa Luces or Saponi or Saura or Sawokli or Sawakola or Sabacola or Sabacola or Savacola or Saxapahaw or Sissipahua or

Shacioes or Seminole or Sewee or Suye or Joye or Xoye or Soya or Shakori or Shoccoree or Stegarake or Stuckanox or Stukanox or Sugeree or Sagarees or Sugaws or Sugar or Succa or Surruque or Suteree or Sitteree or Sutarees or Sataree or Taensa or Talapoosa or Tawasa or Tequesta or Terocodame or Codam or Hieroquodame or Oodame or Perocodame or Teroodame or Timucua or Acuera or Agua Fresca or Aqua Dulce or Freshwater or Arapaha or Cascangue or Icafui or Icafi or Mocama or Tacatacuru or Northern Utina or Ocale or Oconi or Potano or Saturiwa or Tucururu or Tucuru or Yufera or Yui or Ibi or Yustaga or Tiou or Tioux or Tocaste or Tocobaga or Tohome or Tomahitan or Topachula or Tukabatchee or Tuscarora or Tuskegee or Tutelo or Tunica or Tonica or Tonnica or Thonnica or Uzita or Vicela or Viscaynos or Waccamaw or Wateree or Guatari or Watterees or Waxhaw or Waxsaws or Wisack or Wisacky or Weesock or Flathead or Westo or Winyaw or Woccon or Yamasee or Yazoo or Yuchi or Euchee).tw. [Indigenous peoples of the Southeastern Woodlands]

29 (Ak Chin or Southern Athabaskan or Chiricahua Apache or Jicarilla Apache or Lipan Apache or Mescalero Apache or Navajo or Navaho or Dine or San Carlos Apache or Tonto Apache or Western or Coyotero Apache or White Mountain Apache or Aranama or Hanama or Haname or Chaimame or Chariname or Xaraname or Taraname or Coahuiltecan or Cocopa or Comecrudo or Cotoname or Genizaro or Halchidhoma or Hualapai or Havasupai or Hohokam or Karankawa or Kavelchadhom or La Junta or Mamulique or Manso or Maricopa or Mojave or Pima or Pima Bajo or Pueblo people or Ancestral Pueblo or Hano or Hopi or Keres people or Acoma or Cochiti or Laguna or San Felipe or Santa Ana or Santo Domingo or Pueblo or Zia or Nambe or Piro or Pojoaque or San Ildefonso or Tesuque or Santa Clara or Isleta or Picuris or Sandia or Taos or Ysleta del Sur or Tigua or Jemez Pueblo or Tewa or Ohkay Owingeh or Tiwa or Towa or Zuni people or Quechan or Yuma or Quems or Solano or Tamique or Toboso or Tohono Oodham or Qahatika or Tompiro or Ubate or Walapai or Yaqui or Yoreme or Yavapai or Tolkapaya or Western Yavapai or Yavape or Northwestern Yavapai or Kwevkapaya or Southeastern Yavapai or Wipukpa or Northeastern Yavapai).tw. [Indigenous peoples of American Southwest]

30 (Ahtna or Ahtena or Nabesna or Anishinaabe or Oji-Cree or Anishinini or Severn Ojibwa or Ojibwa or Chippewa or Ojibwe or Odawa or Atikamekw or Bearlake or Chipewyan or Cree or Dakelh or Babine or Wetsuweten or Deg Hitan or Deg Xinag or Degexitan or Kaiyuhkhotana or Denaina or Dunneza or Gwichin or Kutchin or Loucheaux or Han or Hare or Holikachuk or Innu or Montagnais or Naskapi or Kaska or Nahane or Kolchan or Upper Kuskokwim or Koyukon or Naskapi or Sekani or Slavey Tagish or Tahltan or Tanana or Tanacross or Tasttine Tlicho or Inland Tlingit or Tsilhqotin or Tutchone or Yellowknives).tw. [Indigenous peoples of the Subarctic]

31 (Aleut or Kalaallit or Inuit or Inupiat or Metis or Yupik).tw. [North American Arctic]

32 (Amuzgo or Chocho or Cocopa or Guarijio or Kikapu or Paipai or Tepehuan or Chontal de Oaxaca or Chatino or Chichimeca Jonaz or Chinantec or Chol or Chontal Maya or Cochimi or Cora or Cuicatec or Huastec or Huave or Huichol or Ixcatec or Kiliwa or Kumeyaay or Lacandon or Matlatzinca or Mayo or Mazahua or Mazatec or Mexicanero or Mixe or Mixtec or Nahua or Pame or Pima Bajo or Popoloca or Purhepecha or Seri or Tarahumara or Tlapanec or Mephaa or Totonac or Trique or Tzeltal or Tzotzil or Yaqui or Yucatec Maya or Zapotec or Zoque).tw. [Mexico]

33 (Ache or Ashaninka or Awa-Guaja or Awa-Kwaiker or Aymara or Ayoreo or Bora or Bororo or Charrua or Chayahuita or Cocama-Cocamilla or Conibo or Embera or Enxet or Jivaroan or Guarani or Karaja or Korubu or Kuna or Makuxi or Mapuche or Matis or Matses or Nukak or Nasa or Secoya or Shipibo or Tapirape or Ticuna or Tukano or Tupi or Urarina or Uwa or Yora or Wichi or Warao or Wayuu or Yanomami or Awajun or Ingano).tw. [South America]

34 (Assyrians or Marsh Dwellers or Madan).tw. [Western Asia]

35 (Oirats or Sartuul or Khoid or Naimans or Mangud or Uyghur or Yaghnobi).tw. [Central Asia]

36 (Ainu or Ryukyuans or Taiwanese aborigines or Amis or Atayal or Bunun or Kavalan or Paiwan or Puyuma or Rukai or Saisiyat or Sakizaya or Seediq or Tao or Thao or Tsou or Truku Salar).tw. [East Asia]
37 (Sakha or Tuvans or Altayans or Buryats or Khakas or Tungus or Sami or Saami).tw. [North Asia]
38 (Aleuts or Alyutors or Chelkans or Chukchis or Chulyms or Chuvans Dolgans or Enets or Yenets or Entsy or Eskimo or Siberian Yupik or Inuit or Evenks or Itelmens or Kamchadals or Kereks or Kets or Khanty or Ostyaks or Koryaks or Kumandins or Mansi or Voguls or Nanai or Negidals or Nenets or Nentsy or Samoyeds or Nganasans or Nivkhs or Orok peopleor or Oroch people or Sami or Lopars or Lapp or Selkups or Shors or Soyots or Taz or Telengits or Teleuts or Tofalars or Tofa or Tubalars or Tozhu or Udege or Ulchs or Veps or Yukaghirs).tw. [Northern indigenous peoples of Russia]
39 (Adivasi or Andamanese or Great Andamanese or Jarawa or Onge or Sentinelese or Bhutia or Lepcha or Kisan Tribals or Nicobari or Shompen or Vedda people or Wanniyala-Aetto or Ladakhi or Kodava people or Toda or Kuruba or Badaga or Kota or Irula or Mech Kachari or Bodo or Naga or Gond or Kalasha of Chitral or Khasi-Jaintia or Tripuri or Raute or Tharu or Giraavaru people or Chakma people or Marma people or Karbi people or Nocte people or Bhil or Dalit).tw. [South Asia]
40 (Idu mishmi or Akha or Degar or Hmong or Karen or Khmuic groups or Khmu or Pray or Mlabri or Lahu or Karbi or Lisu or Negrito or Orang Asli or Tai or Dai or Tai Lu or Tai Dam or Tai Nua or Khun or Phu Thai or Yi or Pribumi or Bajau or Dayak or Igorot or Lumad or Mangyan or Tribes of Palawan or Penan or Batak).tw. [Southeast Asia]
41 (Basques or Crimean Karaites or Crimean Tatars or Izhorians or Komi or Mordvins or Nenets or Sami or Udmurts or Veps).tw. [Europe] (3142)
42 (Evenks or Inuit or Kalaallit or Koryaks or Chukchi or Sami or Yupik or Alutiiq or Yupik or Cupik or Inupiat or Northern Samoyedic peoples or Nenets or Enets or Nganasan or Ugric peoples or Khanty or Mansi or Yukaghirs).tw. [Circumpolar North]
43 (Koori or Koorie or Ngunnawal or Murri or Murrdi or Nyungar or Yamatji or Wangai or Nunga or Anangu or Yapa or Yolngu or Bininj or Tiwi or Anindilyakwa or Palawah or Pallawah or Torres Strait Islanders or Djabugay people).tw. [Indigenous Australians]
44 (Fijian or Papuans or Wopkaimin or Hewa or Kaluli or Dani or Ni-Vanuatu or Malaitan).tw. [Melanesia]
45 (Chamorros or Chuukese or Kiribatese or Kosraean or Marshallese or Nauruan or Pohnpeian or Trukic).tw. [Micronesia]
46 11 or 12 or 13 or 14 or 15 or 16 or 17 or 18 or 19 or 20 or 21 or 22 or 23 or 24 or 25 or 26 or 27 or 28 or 29 or 30 or 31 or 32 or 33 or 34 or 35 or 36 or 37 or 38 or 39 or 40 or 41 or 42 or 43 or 44 or 45
47 limit 46 to animals
48 limit 47 to humans
49 47 not 48
50 46 not 49
51 Information Dissemination/
52 Translational Medical Research/
53 Community-Based Participatory Research/
54 Health Promotion/mt, og [Methods, Organization & Administration]
55 "diffusion of innovation"/
56 "bench to bedside".tw.
57 ("diffusion of innovation" or "implementation of existing research knowledge").tw.
58 "linkage and exchange".tw.
59 "knowledge to action".tw.
60 (community based participatory research or participatory action research).tw.

APPENDIX 1
Effective knowledge translation approaches and practices in Indigenous health research: A systematic review protocol

61 (dissemination and (studies or strategies or research)).tw.
62 mode 2 research.tw.
63 (co-production or coproduction).tw.
64 (co-generation or cogeneration).tw.
65 engaged scholarship.tw.
66 (integrated knowledge or integrated KT or integrated research).tw.
67 (social marketing campaigns or mass media or media campaign).tw.
68 (cultural adaptation or culturally adapted or culturally relevant education or culturally appropriate or cultural brokers).tw.
69 ((knowledge or research or evidence) and (applied health or broker or complex intervention or coordinated or dissemination or exchange or implementation or information or institutionalization or institutionalisation or know-do-gap or broker or diffusion or mobilization or synthesis or to action or to practice or transfer or translation or uptake or user* or utilisation or utilization or policy-relevant or into practice or linkage or uptake or translational)).tw.
70 ((research or evidence or guideline*) adj3 (implementation or utilization or utilisation or diffusion or translation)).tw.
71 51 or 52 or 53 or 54 or 55 or 56 or 57 or 58 or 59 or 60 or 61 or 62 or 63 or 64 or 65 or 66 or 67 or 68 or 69 or 70
72 Health Services Research/
73 exp Program Evaluation/
74 evaluation studies as topic/
75 evaluation studies/
76 "Outcome Assessment (Health Care)"/
77 "Outcome and Process Assessment (Health Care)"/
78 "Process Assessment (Health Care)"/
79 nursing evaluation research/
80 ((Knowledge or research or evidence) adj3 (impact or change or evaluation* or adoption or quality improvement or best practice* or innovation or assessment or outcome* or wise practice*)).tw.
81 (evaluation adj1 (study or studies or framework)).tw.
82 ((program* or service* or outcome* or summative or formative) adj1 evaluation).tw.
83 72 or 73 or 74 or 75 or 76 or 77 or 78 or 79 or 80 or 81 or 82
84 50 and 71 and 83
85 remove duplicates from 84
